# Relationship between tumour PTEN/Akt/COX-2 expression, inflammatory response and survival in patients with colorectal cancer

**DOI:** 10.18632/oncotarget.12134

**Published:** 2016-09-20

**Authors:** Antonia K. Roseweir, Arfon G.M.T. Powell, Lindsay Bennett, Hester C. Van Wyk, James Park, Donald C McMillan, Paul G. Horgan, Joanne Edwards

**Affiliations:** ^1^ Academic Unit of Surgery, School of Medicine, University of Glasgow, Royal Infirmary, Glasgow, United Kingdom; ^2^ Unit of Experimental Therapeutics, Institute of Cancer Sciences, University of Glasgow, Garscube Estate, Glasgow, United Kingdom; ^3^ Division of Cancer and Genetics, Cardiff University, Heath Park, Cardiff, United Kingdom

**Keywords:** colorectal cancer, PTEN, Akt, COX-2, inflammation

## Abstract

In patients with colorectal cancer (CRC), local and systemic inflammatory responses have been extensively reported to associate with cancer survival. However, the specific signalling pathways responsible for inflammatory responses are not clear. The PTEN/Akt pathway is a plausible candidate as it may play a role in mediating inflammation via COX-2, and has been associated with cancer progression. This study therefore examined the relationship between tumour PTEN/Akt/COX-2 expression, inflammatory responses and survival in CRC patients using a tissue microarray.

In 201 CRC patients, activation of tumour-specific PTEN/Akt significantly associated with poorer CSS (12.0yrs v 7.3yrs, *P*=0.032), poorer differentiation (*P*=0.032), venous invasion (*P*=0.008) and peritoneal involvement (*P*=0.004). Patients were stratified for peri-nuclear expression of COX-2 to examine associations with inflammatory responses. In patients with absent peri-nuclear COX-2 expression, activation of tumour-specific PTEN/Akt significantly associated with poorer CSS (11.9yrs v 5.4yrs*, P*=0.001), poorer differentiation (*P*=0.018), venous invasion (*P*=0.003) and peritoneal involvement (*P*=0.001). However, no associations were seen with either the local or systemic inflammatory responses.

In CRC patients, tumour-specific PTEN/Akt pathway activation was significantly associated with poorer CSS, particularly when peri-nuclear COX-2 expression was absent. However, activation of the PTEN/Akt pathway appears not to be responsible for the regulation of inflammatory responses.

## INTRODUCTION

Colorectal cancer (CRC) is the second most common cause of cancer death in Europe [1]. Although outcomes have improved over the past decades, predominantly as a result of improvements in surgical technique and adjuvant/neo-adjuvant therapies, survival still remains poor, with 5-year survival of 50% across all stages of disease [2]. It is clear that the present TNM-based staging of CRC is suboptimal, with a need to identify characteristics pertaining to both the tumour and the host which may not only guide prognosis, but also novel adjuvant therapies.

Local and systemic inflammatory responses have been widely shown to play an active role in tumour development across a wide range of cancers including CRC [3–5]. This is now an area of intense research producing inflammatory-based scoring systems such as the Galon's immunoscore [6], Klintrup-Makinen grade [7] or Glasgow Microenvironment score (GMS),[8] for local inflammation and the modified Glasgow prognostic score (mGPS) [9–11] or neutrophil-lymphocyte ratio (NLR) [12] for systemic inflammation, all of which have prognostic value independent of TNM staging [13]. However, the molecular pathways driving these local and systemic inflammatory responses in CRC are not clear and establishment of the prognostic value of these pathways may provide novel therapeutic targets.

It has long been recognised that the prostaglandin pathway plays an important role when COX-2 is activated by inflammatory stimuli or CRC development. COX-2 is the target for non-steroidal anti-inflammatory drugs (NSAIDs) and these are associated with beneficial treatment in patients with CRC. Cytoplasmic and peri-nuclear COX-2 can regulate the synthesis of prostaglandin E_2_ (PGE_2_), which is commonly upregulated in colon cancer [14]. PGE_2_ promotes inflammation via its receptors (EP1-4) to push T-cell differentiation towards a T-helper 1 (Th1) response, as is commonly seen within the CRC tumour microenvironment [15]. This suggests that COX-2 and PGE_2_ may play important roles in mediating the protective effects of the local inflammatory infiltrate within CRC patients. As well as regulating inflammation, COX-2 mediated synthesis of PGE_2_ has also been shown to regulate proliferation and migration of CRC cell lines [9, 16].

It has recently been delineated that the PTEN/Akt pathway can regulate expression of COX-2 and both phosphatase and tensin homolog (PTEN) and Akt are proposed to play an important role in cancer progression as well as inflammatory responses [17, 18]. For example, Akt can regulate migration of neutrophils, macrophages and CD8+ T-cells to the site of infection [19] and PTEN plays a role in suppressing Treg cell generation and function to remodel the tumour microenvironment [20–22]. This suggests that the PTEN/Akt pathway may regulate inflammatory responses via modulation of COX-2 expression. The PTEN/Akt cascade has also been shown to be dysregulated in a variety of cancers [23–26].

However, the relationship between tumour PTEN/Akt/COX-2 expression and inflammatory responses in patients with CRC is not well defined, and most publications have focused on *in vitro* data [27]. Therefore, the aim of the present study was to examine the relationship between tumour PTEN/Akt/COX-2 expression, inflammatory responses and survival in patients with CRC.

## RESULTS

### The PTEN/Akt pathway, cancer-specific survival and clinicopathological characteristics in CRC patients

A total of 201 patients, who underwent an elective, potentially curative resection of stage I-III CRC and had a valid score for all three assessed proteins were included in the study (Supplementary Table 1). Almost two thirds of patients were 65 or older at the time of surgery and just over half were male. Two thirds of patients underwent resection for colon cancer. Fourteen patients (7%) had pathological confirmation of stage I disease, whereas 96 (48%) and 91 (45%) patients had stage II and stage III disease respectively. Twenty-seven patients (14%) had MMR deficient CRC, and fifty-six patients (28%) received adjuvant chemotherapy. The median follow-up of survivors was 11.2 years (range 6.2-16.0yrs) with 62 cancer-associated deaths and 55 non-cancer deaths.

The histoscore for Akt^473^ phosphorylation ranged from 0-154 within the cytoplasm and from 0-24 at the membrane. PTEN expression ranged from 0-210 in the cytoplasm and 0-63 at the membrane (Supplementary Figure 1). Scores where split by the median into high and low expression for each protein at each cellular location. Neither cytoplasmic nor membrane Akt^473^ were associated with cancer-specific survival (CSS, Table 1). Similarly, neither cytoplasmic nor membrane PTEN were associated with CSS (Table 1).

**Table 1:** PTEN/Akt^473^/COX-2 expression and survival in patients undergoing elective, potentially curative resection of colorectal cancer (n=201)

	*N (%)*	10yr-CSS % (SE^a^)	Univariate HR^b^ (95% CI^c^)	*P*
**Cytoplasmic Akt^473^** ** Low expression** ** High expression**	95 (47) 106 (53)	70 (5) 65 (5)	1.15 (0.70-1.89)	0.592
**Membrane Akt^473^** ** Low expression** ** High expression**	160 (80) 41 (20)	70 (4) 56 (9)	1.36 (0.76-2.45)	0.296
**Cytoplasmic PTEN** ** Low expression** ** High expression**	102 (51) 109 (49)	67 (5) 69 (5)	0.96 (0.58-1.58)	0.862
**Membrane PTEN** ** Low expression** ** High expression**	123 (61) 78 (39)	65 (5) 72 (6)	0.71 (0.41-1.20)	0.194
**Combined cPTEN/mAkt^473^ (simple model)** ** High PTEN/Low Akt^473^** ** High PTEN/High Akt^473^** ** Low PTEN/Low Akt^473^** ** Low PTEN/High Akt^473^**	77 (38) 83 (41) 22 (11) 19 (10)	69 (6) 71 (5) 60 (17) 47 (11)	1.14 (0.88-1.48)	0.159
**Combined cPTEN/mAkt^473^ (off/on model)** ** All others (OFF)** ** Low PTEN/High Akt^473^ (ON)**	182 (90) 19 (10)	70 (4) 47 (11)	2.06 (1.05-4.07)	**0.032**
**cPTEN/mAkt^473^ off/on model (No nuclear COX-2; n=158)** ** All others (OFF)** ** Low PTEN/High Akt^473^ (ON)**	142 (90) 15 (10)	69 (4) 33 (12)	3.04 (1.51-6.10)	**0.001**
**cPTEN/mAkt^473^ off/on model (with nuclear COX-2; n=45)** ** All others (OFF)** ** Low PTEN/High Akt^473^ (ON)**	40 (91) 4 (9)	72 (7) 100 (0)	0.42 (0.00-185.51)	0.251

a SE= Standard Error,

b HR= Hazard Ratio,

c CI= Confidence Interval.

**Figure 1:**
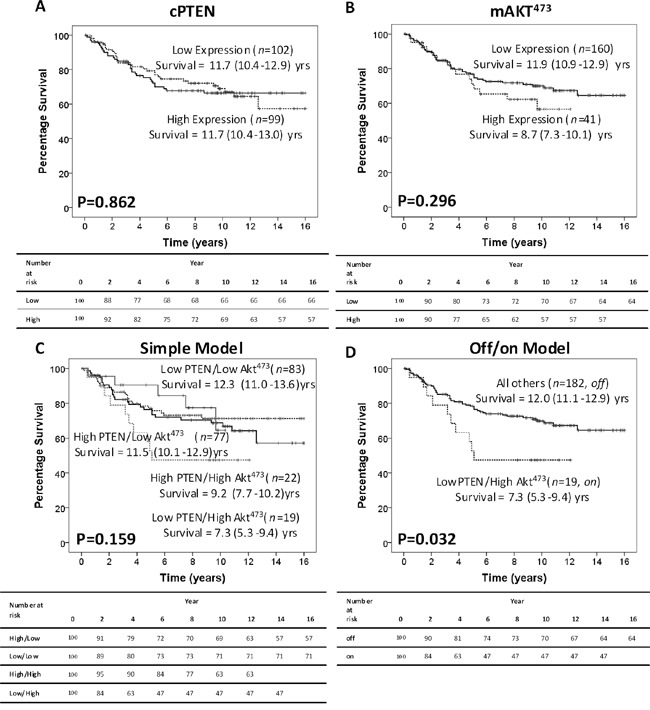
Intra-tumour activation of the PTEN/Akt pathway is associated with cancer-specific survival in CRC patients **A.** Kaplan Meier curve showing that in 201 CRC patients, cPTEN does not associate with CSS. **B.** Kaplan Meier curve showing that in 201 CRC patients, phosphorylation of mAkt^473^ does not associate with CSS. **C.** Kaplan Meier curves show that in 201 CRC patients, the simple model shows a slight trend towards poorer CSS. **D.** Whereas when cPTEN and mAkt^473^ were combined using the off/on model, there is a significant association with poorer CSS.

Although neither cytoplasmic PTEN (cPTEN) expression (main location for PTEN inhibiting Akt activation) nor membrane Akt^473^ (mAkt^473^) phosphorylation (main location for Akt activation) was associated with CSS alone (Figure 1A, 1B), when considered together to make a cumulative prognostic score, were associated with CSS. cPTEN and mAkt^473^ were combined to make a cumulative prognostic score using two different models, 1) simple model: High cPTEN/low mAkt^473^, both high, both low or low cPTEN/high mAkt^473^, and an off/on model of pathway activation: Off= High cPTEN/Low mAkt^473^ or High cPTEN/High mAkt^473^ or Low cPTEN/Low mAkt^473^ and On= Low cPTEN/High mAkt^473^. The simple model did not associated with CSS (Figure 1C), however, the off/on model was significantly associated with poorer CSS (12.0yrs v 7.3yrs, *P*=0.032 (HR 2.06), Figure 1D). The off/on model was also associated with high risk pathological characteristics (Table 2), increased t-stage (*P*=0.030), poorer differentiation (*P*=0.032), venous invasion (*P*=0.008) and peritoneal involvement (*P*=0.004). No association with age, sex, site or inflammatory responses was observed.

**Table 2:** Relationship between cPTEN/mAkt^473^/nCOX-2 expression, clinicopathological characteristics and inflammatory responses in patients undergoing elective, potentially curative resection of colorectal cancer (n=201)

	cPTEN/mAkt^473^			Absent nCOX-2 cPTEN/mAkt^473^		
	OFF (n=182)	ON (n=19)	*P*	OFF (n=142)	ON (n=15)	*P*
**Clinicopathological Characteristics**						
**Age** ** <65** ** >65**	73 (40) 109 (60)	8 (42) 11 (58)	0.866	50 (35) 92 (65)	7 (47) 8 (53)	0.380
**Sex** ** Female** ** Male**	87 (48) 95 (52)	9 (47) 10 (53)	0.971	67 (47) 75 (53)	7 (47) 8 (53)	0.970
**Adjuvant** ** No** ** Yes**	132 (73) 50 (27)	13 (68) 6 (32)	0.704	105 (74) 37 (26)	10 (67) 5 (33)	0.545
**Tumour site** ** Colon** ** Rectum**	121 (66) 61 (34)	12 (63) 7 (37)	0.771	90 (63) 52 (37)	10 (67) 5 (33)	0.801
**T stage** ** 1** ** 2** ** 3** ** 4**	8 (4) 14 (8) 119 (65) 41 (23)	0 (0) 1 (6) 9 (47) 9 (47)	**0.030**	6 (4) 13 (10) 93 (65) 30 (21)	0 (0) 1 (7) 6 (40) 8 (53)	**0.022**
**N stage** ** 1** ** 2** ** 3**	103 (56) 63 (35) 16 (9)	7 (37) 10 (53) 2 (10)	0.174	80 (56) 51 (36) 11 (8)	5 (33) 8 (54) 2 (13)	0.103
**Differentiation** ** Mod/well** ** Poor**	164 (90) 18 (10)	14 (74) 5 (26)	**0.032**	131 (93) 11 (7)	11 (73) 4 (27)	**0.018**
**Venous invasion** ** Absent** ** Present**	123 (68) 59 (32)	7 (37) 12 (63)	**0.008**	94 (66) 48 (34)	4 (27) 11 (73)	**0.003**
**Margin involvement** ** No** ** Yes**	176 (97) 6 (3)	18 (95) 1 (5)	0.656	136 (96) 6 (4)	14 (94) 1 (6)	0.663
**Peritoneal involvement** ** No** ** Yes**	141 (77) 41 (23)	9 (47) 10 (53)	**0.004**	112 (79) 30 (21)	6 (40) 9 (60)	**0.001**
**Mismatch repair status** ** Competent** ** Deficient**	158 (87) 24 (13)	16 (84) 3 (16)	0.759	126 (89) 16 (11)	13 (87) 2 (13)	0.819
**Proliferation Index** ** Low** ** High**	67 (37) 115 (63)	5 (26) 14 (74)	0.362	51 (36) 91 (64)	5 (33) 10 (67)	0.855
**Necrosis** ** Low** ** High**	113 (62) 69 (38)	9 (47) 10 (53)	0.214	91 (64) 51 (36)	7 (47) 8 (53)	0.187
**Tumour stroma percentage (n=182)** ** Low** ** High**	130 (78) 36 (22)	10 (63) 6 (37)	0.152	105 (81) 24 (19)	8 (62) 5 (38)	0.091
**Tumour Budding (n=186)** ** No** ** Yes**	117 (69) 53 (31)	9 (56) 7 (44)	0.304	90 (68) 42 (32)	6 (46) 7 (54)	0.109
**Inflammatory Characteristics**						
**Klintrup-Makinen grade** ** Strong** ** Weak**	65 (36) 117 (64)	7 (37) 12 (63)	0.936	51 (36) 90 (64)	6 (40) 9 (60)	0.770
**mGPS** ** 0** ** 1** ** 2**	104 (57) 55 (30) 23 (13)	14 (74) 5 (26) 0 (0)	0.081	83 (58) 44 (31) 15 (11)	11 (73) 4 (27) 0 (0)	0.160
**NPS (n=114)** ** 0** ** 1** ** 2**	67 (68) 31 (31) 1 (1)	11 (73) 3 (20) 1 (7)	0.224	59 (70) 24 (29) 1 (1)	9 (69) 3 (23) 1 (8)	0.296
**NLR (n=177)** ** ≤ 5** > **5**	124 (78) 35 (22)	15 (83) 3 (17)	0.601	106 (84) 20 (16)	12 (86) 2 (14)	0.877

### The PTEN/Akt pathway, peri-nuclear COX-2 and cancer-specific survival in CRC patients

**Figure 2:**
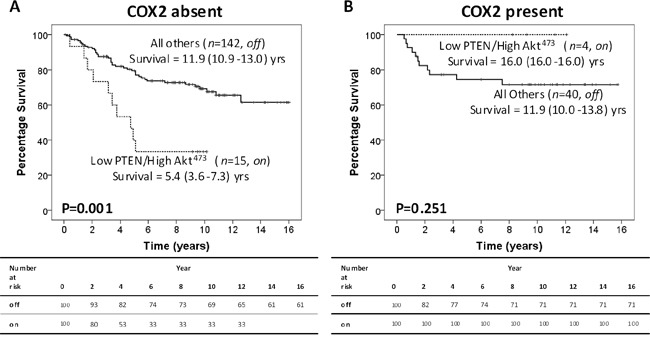
Peri-nuclear COX-2 stratifies CRC patients with an active intra-tumour PTEN/Akt pathway **A.** Kaplan Meier curve showing that in 157 CRC patients with absent pnCOX-2 expression, the off/on model significantly associates with poorer CSS. **B.** Kaplan Meier curve showing that in 44 CRC patients with pnCOX-2 expression, the off/on model does not associate with CSS.

As COX-2 is a potential downstream target of the PTEN/Akt pathway in cancer and has a role in inflammation, peri-nuclear tumour cell expression was assessed to investigate if COX-2 could provide a link between activation of tumour PTEN/Akt and inflammation. Peri-nuclear COX-2 (pnCOX-2) expression was assessed as absent or present (Supplementary Figure 1). When the off/on model was stratified by pnCOX-2 absence or presence; in patients with absent pnCOX-2 expression, the off/on model was associated with significantly poorer CSS (11.9yrs v 5.4yrs, *P*=0.001 (HR 3.04); Figure 2A). The 10yr-CSS rates were 69% and 33% for off and on, respectively (*P*=0.005, Table 1). In patients with pnCOX-2 expression, there was no significant association of the off/on model with CSS (11.9yrs v 16.0yrs, *P*=0.251 (HR 0.42), Figure 2B). The 10yr-CSS rates were 71% (off) and 100% (on) in these patients (*P*=0.222, Table 1).

### The PTEN/Akt pathway, peri-nuclear COX-2 and clinicopathological characteristics in CRC patients

**Figure 3:**
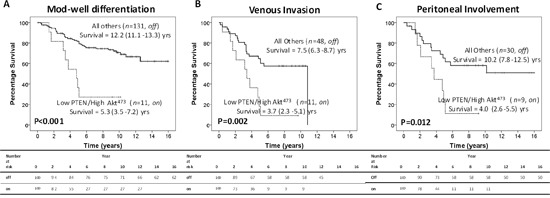
Intra-tumour activation of the PTEN/Akt/COX-2 pathway modulates CRC progression and invasive capacity In patients with absent pnCOX-2 expression, the off/on model significantly associates with poorer CSS in patients with **A.** moderate to well differentiation, **B.** venous invasion and **C.** peritoneal involvement.

It is only when pnCOX-2 expression is absent that the off/on model continues to associate with poorer CSS. In these patients, the off/on model is also more strongly associated with the high risk pathological characteristics (Table 2), increased t-stage (*P*=0.022), poorer differentiation (*P*=0.018), venous invasion (*P*=0.003) and peritoneal involvement (*P*=0.001). When patients are stratified for these factors, the off/on model is still significantly associated with poorer CSS in patients with moderate to well differentiation (12.2yrs v 5.3yrs, *P*<0.001, Figure 3A), venous invasion (7.5yrs v 3.7yrs, *P*=0.002, Figure 3B) and peritoneal involvement (10.2yrs v 4.0yrs, *P*=0.012, Figure 3C). However, no association was seen with either local or systemic inflammatory responses (Table 2).

### Is the PTEN/Akt pathway an independent prognostic factor for CRC?

Since the off/on model had a significant association with poorer CSS in patients with absent pnCOX-2 expression, this model was taken into univariate and multivariate analysis (Table 3). On univariate analysis, sex (*P*=0.014), TNM stage (*P*<0.001), venous invasion (*P*<0.001), margin involvement (*P*=0.005), peritoneal involvement (*P*=0.002), proliferation index (*P*=0.024), TSP (*P*<0.001), tumour budding (*P*<0.001) and KM Grade (*P*=0.005) were significantly associated with poorer CSS (Table 3).

**Table 3:** Univariate and multivariate survival analysis for patients with absent nCOX-2 expression undergoing elective, potentially curative resection of colorectal cancer (n=157)

	Univariate HR^a^ (95% CI^b^)	*P*	Multivariate HR^a^ (95% CI^b^)	*P*
**Clinicopathological Characteristics**				
**Age (<65/>65)**	0.77 (0.44-1.35)	0.362	-	-
**Sex (Female/Male)**	2.10 (1.16-3.80)	**0.014**	1.38 (0.69-2.84)	0.385
**Adjuvant Therapy (No/Yes)**	1.35 (0.75-2.45)	0.320	-	-
**Tumour Site (Colon/Rectum)**	1.19 (0.68-2.10)	0.546	-	-
**TNM Stage (1/2/3)**	2.93 (1.71-5.02)	**<0.001**	2.22 (1.08-4.53)	**0.030**
**Differentiation (Moderate or well/Poor)**	2.22 (1.00-4.94)	0.051	-	-
**Venous Invasion (Absent/Present)**	3.31 (1.86-5.89)	**<0.001**	2.57 (1.22-5.38)	**0.013**
**Margin Involvement (No/Yes)**	3.76 (1.48-9.52)	**0.005**	3.56 (1.29-9.83)	**0.014**
**Peritoneal Involvement (No/Yes)**	2.44 (1.39-4.27)	**0.002**	1.02 (0.49-2.14)	0.958
**Mismatch Repair Status (Competent/Deficient)**	0.84 (0.33-2.11)	0.705	-	-
**Proliferation Index (Low/High)**	0.53 (0.30-0.92)	**0.024**	0.57 (0.30-1.10)	0.093
**Necrosis (Low/High)**	1.32 (0.75-2.33)	0.335	-	-
**Tumour Stroma Percentage (<50%/>50%)**	3.68 (2.01-6.74)	**<0.001**	1.92 (0.89-4.11)	0.094
**Tumour Budding (No/Yes)**	3.76 (2.05-6.88)	**<0.001**	2.44 (1.07-5.55)	**0.034**
**Inflammatory Characteristics**				
**Klintrup-Makinen Grade (Strong/Weak)**	2.68 (1.34-5.36)	**0.005**	3.00 (1.34-6.73)	**0.008**
**mGPS (0/1/2)**	1.12 (0.74-1.70)	0.596	-	-
**NPS (0/1/2)**	1.86 (0.95-3.64)	0.068	-	-
**NLR (<5/>5)**	0.81 (0.32-2.06)	0.663	-	**-**
**PTEN/Akt Pathway**				
**cPTEN/mAkt^473^ off/on model (OFF/ON)**	3.04 (1.51-6.10)	**0.001**	1.51 (0.62-3.69)	0.369

a **HR= Hazard Ratio,**

a **CI= Confidence Interval.**

On multivariate survival analysis including the off/on model; TNM stage (*P*=0.030), venous invasion (*P*=0.013), margin involvement (*P*=0.014), tumour budding (*P*=0.034) and KM Grade (*P*=0.008) were independent prognostic factors, with proliferation index (*P*=0.093) and TSP (*P*=0.094) showing trends towards independence (Table 3). However, the PTEN/Akt pathway does not appear to be independently prognostic in these patients (*P*=0.369).

## DISCUSSION

The results of this study show that although the PTEN/Akt pathway is active in patients with CRC and is associated with poorer CSS, this pathway appears not to modulate local or systemic inflammatory responses. Indeed, the results of the present study suggest this pathway would be important within the tumour since it was associated with poorer differentiation, venous invasion and peritoneal involvement. Finally, this study confirms the importance of examining the PTEN/Akt pathway as a whole and not the individual components in isolation for patients with CRC.

The results within the present study are consistent with other studies that have suggested that activation of Akt can promote tumorigenesis by modulating tumour differentiation and invasion. Akt is a serine/threonine-specific kinase with three isoforms, the ubiquitously expressed Akt1 and Akt2 plus the neuronal-specific Akt3. Akt1 is the most prominent and plays a role in many cellular processes which are important for cancer progression. Effects of Akt1 on differentiation and invasion are now starting to emerge in a variety of cancers. In CRC, *in vitro* studies have proposed that Akt1 can inhibit cell growth and promote metastatic invasion by inducing epithelial to mesenchymal transition (EMT) [28, 29]. In the present study, there was a trend towards an association between tumour budding and patients with an active pathway. Tumour budding is suggested to be a marker for EMT. However, little other *in vivo* evidence exists to support this theory in CRC. There are, however, studies in patients with other cancers types. In prostate cancer patients, phosphorylated Akt1 is more highly expressed at the membrane of high grade, poorly differentiated tumours [30]. Akt1 has been shown to be an essential regulator of mammary cell differentiation in breast cancer via regulation of the STAT5 pathway [31]. Riggio et al. also showed that Akt1 regulates epithelial breast cancer differentiation via positive regulation of cytokeratin-8 and basement membrane formation. Furthermore, they suggested that Akt1 could affect tumour cell invasion via phosphorylation of focal adhesion kinase (FAK) [32]. The results of these studies propose an intra-tumour role for activated Akt1 as an important regulator of tumour progression and metastasis.

Loss of PTEN is also hallmark of many cancers including CRC (particularly in MSI-CRC), causing hyper-activation of the survival promoting Akt pathway and as this study shows poorer prognosis for patients [33–35]. In CRC patients, several groups have shown that loss of PTEN correlates with local recurrence, advanced stage, lymph node metastasis and poorer CSS, suggesting a link between PTEN loss and an invasive CRC phenotype [36–38]. This invasive phenotype is also seen *in vitro*, where PTEN knock-down potentiates the invasiveness of HCT116 colorectal cancer spheroidal cells through a 3D extracellular matrix [39]. Similar effects are also seen in MKN-28 gastric cancer cells, where PTEN inhibits tumour cell growth and invasion via the downregulation of FAK expression [40].

However, within the present study associations with CSS, invasion and differentiation were only present when both Akt and PTEN are considered together. This is perhaps not unexpected as PTEN antagonizes phosphorylation of Akt when it resides in the cytoplasm and is therefore required to be downregulated in order for Akt to be activated [41]. Therefore, optimal activation of the pathway occurs when high Akt levels are present in the membrane and low PTEN levels are present in the cytoplasm, it is for this reason that these cellular locations were chosen for the models. This study also highlighted that looking at the whole pathway as active or inactive as for the off/on models is more relevant than simply combining low/high expression of the two proteins as done in the simple model. This study therefore proposes that looking at PTEN and Akt in isolation within CRC patients may not be the best approach and that considering the pathway as a whole may be crucial to understanding their function in this cancer.

Furthermore, the data indicates that the PTEN/Akt pathway may be modulating pnCOX-2 expression to promote tumorigenesis. Since, the data does not reveal any associations with either local or systemic inflammatory responses; this suggests that pnCOX-2 has a role independent of inflammation in CRC. Furthermore, as it is patients with absent pnCOX-2 expression that have the worse prognosis, this proposes that pnCOX-2 can act as a tumour suppressor in CRC. It has been reported that COX-2 has a separate role as a transcription factor in the nucleus [42, 43] however the target genes for COX-2 have not been delineated. It has also been shown that COX-2 may have tumour suppressive effects in mice where COX-2 has been overexpressed or synthetic PGE2 administered [44, 45].

In conclusion, the results of this study show the PTEN/Akt pathway plays a role in CRC progression and is associated with a poorer prognosis for patients. However, this pathway appears not to be responsible for modulation of local or systemic inflammatory responses. The data does indorse that examining the pathway as a whole, such as with the off/on model may be more beneficial than looking at the individual components. The results from the off/on model suggest that the effects of the pathway are targeted to the tumour itself to modulate cancer progression by regulating pnCOX-2 expression to promote cellular differentiation and invasion. However, further work is still needed to fully understand the role of this pathway in CRC.

## MATERIALS AND METHODS

### Patients

Patients were identified from a prospectively collected and maintained database of CRC resections performed in a single surgical unit in Glasgow Royal Infirmary. For the present study, 201 patients who between 1997 and 2007 had undergone an elective, potentially curative resection for stage I-III CRC and were contained within a previously constructed tissue microarray (TMA) were included. Resection was considered curative on the basis of pre-operative computed tomography and intra-operative findings. Patients who received neoadjuvant therapy, or had died within 30 days of surgery were excluded. Ethical approval was obtained from the West of Scotland Research Ethics Committee.

### Clinicopathological characteristics

Tumours were staged using the fifth edition of the AJCC/UICC-TNM staging system[46]. The presence of venous invasion was assessed using elastica staining. Following surgery, patients with stage III or high-risk stage II disease and without significant co-morbid disease precluding adjuvant treatment were considered for 5-fluorouracil-based chemotherapy. Patients were followed up and date and cause of death were crosschecked with the cancer registration system and the Registrar General (Scotland). Cancer-specific survival (CSS) was measured from date of surgery until date of death from CRC.

The presence of tumour necrosis and tumour stroma percentage (TSP) were assessed as previously described [47]. Briefly using haematoxylin & eosin (H&E)-stained sections of the deepest point of invasion, necrosis was graded as either low-grade (focal or absent) or high-grade (moderate or extensive) and TSP was either graded as low (≤50%) or high (>50%). Mismatch repair (MMR) status was assessed as previously described [8]. Ki67 proliferation index were previously established in this cohort.

The local inflammatory cell infiltrate was assessed using the Klintrup-Mäkinen (KM) grade as previously described [48]. Briefly, using full H&E sections of the deepest point of invasion, the inflammatory cell infiltrate at the invasive margin was graded as either low-grade (no increase or mild/patchy increase in inflammatory cells) or high-grade (prominent inflammatory reaction forming a band at the invasive margin, or florid cup-like infiltrate at the invasive edge with destruction of cancer cell islands). Neutrophil, platelet and lymphocyte counts were previously established in this cohort and used to generate the neutrophil-platelet score (NPS) and neutrophil-lymphocyte ratio (NLR).

Serum C-reactive protein (CRP) and albumin were recorded prospectively and measured within 30 days prior to surgery. The pre-operative systemic inflammatory response was defined using the modified Glasgow prognostic score (mGPS). The mGPS was calculated as previously described [13]; patients with CRP ≤10 mg/L were allocated a score of 0, patients with CRP >10 mg/L a score of 1, and patients with CRP >10 mg/L and albumin <35g/L were allocated a score of 2.

### Immunohistochemistry

Immunohistochemical expression of PTEN, Akt^473^ and COX-2 was carried out using a previously constructed CRC TMA [49]. Sections were dewaxed in xylene then rehydrated using graded alcohols. Antigen retrieval for Akt^473^was performed using TE buffer pH9 at 96°C for 20 minutes, PTEN in citrate buffer pH6 under pressure for 5 minutes and COX-2 in TE buffer pH9 under pressure for 5 minutes before cooling for 20 minutes. Endogenous peroxidase activity was blocked using 3% hydrogen peroxide for 10 minutes. 5% horse serum (PTEN/Akt^473^) or 20% goat serum (COX-2) was applied for 20 minutes at room temperature as a blocking solution. TMA sections were incubated overnight at 4°C with primary AKT^473^or PTEN (Cell Signaling Technologies, US) antibody at a concentration of 4 ug/ml and 1 ug/ml, respectively or for 2 hours at room temperature with primary COX-2 antibody (Abcam, UK) at 1:1000 dilution before washing the sections in TBS. Envision (Dako) was added to the sections for 30 minutes at room temperature before washing in TBS. DAB substrate was added for five minutes until colour developed before washing in running water for ten minutes. Slides were then counterstained in haematoxylin for 60 seconds and blued with Scotts' tap water before being dehydrated through a series of graded alcohols. Cover slips were applied using distrene, plasticizer, xylene (DPX).

### Scoring

Stained TMA sections were scanned using a Hamamatsu NanoZoomer (Welwyn Garden City, Hertfordshire, UK) at x20 magnification and visualized on Slidepath Digital Image Hub (Leica Biosystems, Milton Keynes, UK). Assessment of Akt^473^and PTEN expression was performed by a single examiner (A.K.R) blinded to clinical data at x20 magnification (total magnification x400) using the weighted histoscore. The weighted histoscore is calculated as follows: 0x% not stained + 1x% weakly stained + 2x% moderately stained + 3x% strongly stained. This gives a range of scores from 0 to 300 and is calculated individually for membrane and cytoplasmic staining. As COX-2 was observed in the peri-nuclear region, a weighted histoscore could not be applied, therefore peri-nuclear COX-2 data was assessed by a single observer (L.B) and absence or presence of expression was noted. To ensure reproducibility, 10% of tumours were co-scored by a co-investigator (J.E.); the intraclass correlation coefficient was 0.960 for PTEN and 0.882 for Akt^473^.

### Statistical analysis

Only patients with a score for all three proteins were included in the analysis. Patients were divided into quartiles and the median (low/high) proven to be the most appropriate cut-off for analysis for PTEN and Akt^473^. The relationship between clinicopathological characteristics and protein expression was examined using the chi-square test for linear trend. The relationship between expression and CSS was examined using Kaplan-Meier method. The log rank test was utilized to compare significant differences between subset groups using univariate analysis. Multivariate cox regression analysis was performed to identify those factors that were independently associated with CSS. A *P*-value <0.05 was considered statistically significant. All analyses were performed using SPSS version 22.0 (IBM SPSS).

## SUPPLEMENTARY FIGURE AND TABLE



## References

[1] Ferlay J, Steliarova-Foucher E, Lortet-Tieulent J, Rosso S, Coebergh JW, Comber H, Forman D, Bray F (2013). Cancer incidence and mortality patterns in Europe: estimates for 40 countries in 2012. European journal of cancer.

[2] Oliphant R, Nicholson GA, Horgan PG, Molloy RG, McMillan DC, Morrison DS, West of Scotland Colorectal Cancer Managed Clinical N (2013). Deprivation and colorectal cancer surgery: longer-term survival inequalities are due to differential postoperative mortality between socioeconomic groups. Annals of surgical oncology.

[3] McAllister SS, Weinberg RA (2014). The tumour-induced systemic environment as a critical regulator of cancer progression and metastasis. Nat Cell Biol.

[4] Diakos CI, Charles KA, McMillan DC, Clarke SJ (2014). Cancer-related inflammation and treatment effectiveness. Lancet Oncol.

[5] Hanahan D, Coussens LM (2012). Accessories to the crime: functions of cells recruited to the tumor microenvironment. Cancer Cell.

[6] Pages F, Galon J, Dieu-Nosjean MC, Tartour E, Sautes-Fridman C, Fridman WH (2010). Immune infiltration in human tumors: a prognostic factor that should not be ignored. Oncogene.

[7] Klintrup K, Makinen JM, Kauppila S, Vare PO, Melkko J, Tuominen H, Tuppurainen K, Makela J, Karttunen TJ, Makinen MJ (2005). Inflammation and prognosis in colorectal cancer. European journal of cancer.

[8] Park JH, McMillan DC, Powell AG, Richards CH, Horgan PG, Edwards J, Roxburgh CS (2014). Evaluation of a Tumor Microenvironment-Based Prognostic Score in Primary Operable Colorectal Cancer. Clin cancer res.

[9] Buchanan FG, Wang D, Bargiacchi F, DuBois RN (2003). Prostaglandin E2 regulates cell migration via the intracellular activation of the epidermal growth factor receptor. J Biol Chem.

[10] McMillan DC (2013). The systemic inflammation-based Glasgow Prognostic Score: a decade of experience in patients with cancer. Cancer Treat Rev.

[11] Guthrie GJ, Roxburgh CS, Farhan-Alanie OM, Horgan PG, McMillan DC (2013). Comparison of the prognostic value of longitudinal measurements of systemic inflammation in patients undergoing curative resection of colorectal cancer. Br J Cancer.

[12] Guthrie GJ, Charles KA, Roxburgh CS, Horgan PG, McMillan DC, Clarke SJ (2013). The systemic inflammation-based neutrophil-lymphocyte ratio: experience in patients with cancer. Crit Rev Oncol Hematol.

[13] Park JH, Watt DG, Roxburgh CS, Horgan PG, McMillan DC (2015). Colorectal Cancer, Systemic Inflammation, and Outcome: Staging the Tumor and Staging the Host. Ann Surg.

[14] Claria J (2003). Cyclooxygenase-2 biology. Curr Pharm Des.

[15] Ricciotti E, FitzGerald GA (2011). Prostaglandins and inflammation. Arterioscler Thromb Vasc Biol.

[16] Sobolewski C, Cerella C, Dicato M, Ghibelli L, Diederich M (2010). The role of cyclooxygenase-2 in cell proliferation and cell death in human malignancies. Int J Cell Biol.

[17] Xia S, Zhao Y, Yu S, Zhang M (2010). Activated PI3K/Akt/COX-2 pathway induces resistance to radiation in human cervical cancer HeLa cells. Cancer Biother Radiopharm.

[18] St-Germain ME, Gagnon V, Mathieu I, Parent S, Asselin E (2004). Akt regulates COX-2 mRNA and protein expression in mutated-PTEN human endometrial cancer cells. Int J Oncol.

[19] Weichhart T, Saemann MD (2008). The PI3K/Akt/mTOR pathway in innate immune cells: emerging therapeutic applications. Ann Rheum Dis.

[20] Sharma MD, Shinde R, McGaha TL, Huang L, Holmgaard RB, Wolchok JD, Mautino MR, Celis E, Sharpe AH, Francisco LM, Powell JD, Yagita H, Mellor AL, Blazar BR, Munn DH (2015). The PTEN pathway in T is a critical driver of the suppressive tumor microenvironment. Sci Adv.

[21] Crellin NK, Garcia RV, Levings MK (2007). Altered activation of AKT is required for the suppressive function of human CD4+CD25+ T regulatory cells. Blood.

[22] Sauer S, Bruno L, Hertweck A, Finlay D, Leleu M, Spivakov M, Knight ZA, Cobb BS, Cantrell D, O'Connor E, Shokat KM, Fisher AG, Merkenschlager M (2008). T cell receptor signaling controls Foxp3 expression via PI3K, Akt, and mTOR. Proc Natl Acad Sci U S A.

[23] Sasaki T, Yamashita Y, Kuniyasu H (2015). AKT plays a crucial role in gastric cancer. Oncol Lett.

[24] de Araujo WM, Robbs BK, Bastos L, de Souza WF, Vidal-Cabral F, Viola JP, Morgado-Diaz JA (2015). PTEN Overexpression Cooperates With Lithium to Reduce the Malignancy and to Increase Cell Death by Apoptosis Via PI3K/Akt Suppression in Colorectal Cancer Cells. J Cell Biochem.

[25] Ali A, Mishra PK, Sharma S, Arora A, Saluja SS (2015). Effects of PTEN gene alteration in patients with gallbladder cancer. Cancer Genet.

[26] Fresno Vara JA, Casado E, de Castro J, Cejas P, Belda-Iniesta C, Gonzalez-Baron M (2004). PI3K/Akt signalling pathway and cancer. Cancer Treat Rev.

[27] Zhang LL, Mu GG, Ding QS, Li YX, Shi YB, Dai JF, Yu HG (2015). Phosphatase and Tensin Homolog (PTEN) Represses Colon Cancer Progression through Inhibiting Paxillin Transcription via PI3K/AKT/NF-kappaB Pathway. J Biol Chem.

[28] Suman S, Kurisetty V, Das TP, Vadodkar A, Ramos G, Lakshmanaswamy R, Damodaran C (2014). Activation of AKT signaling promotes epithelial-mesenchymal transition and tumor growth in colorectal cancer cells. Mol Carcinog.

[29] Ericson K, Gan C, Cheong I, Rago C, Samuels Y, Velculescu VE, Kinzler KW, Huso DL, Vogelstein B, Papadopoulos N (2010). Genetic inactivation of AKT1, AKT2, and PDPK1 in human colorectal cancer cells clarifies their roles in tumor growth regulation. Proc Natl Acad Sci U S A.

[30] Malik SN, Brattain M, Ghosh PM, Troyer DA, Prihoda T, Bedolla R, Kreisberg JI (2002). Immunohistochemical demonstration of phospho-Akt in high Gleason grade prostate cancer. Clinical cancer research.

[31] Chen CC, Boxer RB, Stairs DB, Portocarrero CP, Horton RH, Alvarez JV, Birnbaum MJ, Chodosh LA (2010). Akt is required for Stat5 activation and mammary differentiation. Breast Cancer Res.

[32] Riggio M, Polo ML, Blaustein M, Colman-Lerner A, Luthy I, Lanari C, Novaro V (2012). PI3K/AKT pathway regulates phosphorylation of steroid receptors, hormone independence and tumor differentiation in breast cancer. Carcinogenesis.

[33] Stone L (2015). Prostate cancer: PTEN loss and PSGR overexpression promote cancer progression. Nat Rev Urol.

[34] Beg S, Siraj AK, Prabhakaran S, Jehan Z, Ajarim D, Al-Dayel F, Tulbah A, Al-Kuraya KS (2015). Loss of PTEN expression is associated with aggressive behavior and poor prognosis in Middle Eastern triple-negative breast cancer. Breast Cancer Res Treat.

[35] Lin PC, Lin JK, Lin HH, Lan YT, Lin CC, Yang SH, Chen WS, Liang WY, Jiang JK, Chang SC (2015). A comprehensive analysis of phosphatase and tensin homolog deleted on chromosome 10 (PTEN) loss in colorectal cancer. World J Surg Oncol.

[36] Li XH, Zheng HC, Takahashi H, Masuda S, Yang XH, Takano Y (2009). PTEN expression and mutation in colorectal carcinomas. Oncol Rep.

[37] Sawai H, Yasuda A, Ochi N, Ma J, Matsuo Y, Wakasugi T, Takahashi H, Funahashi H, Sato M, Takeyama H (2008). Loss of PTEN expression is associated with colorectal cancer liver metastasis and poor patient survival. BMC Gastroenterol.

[38] Lin MS, Huang JX, Chen WC, Zhang BF, Fang J, Zhou Q, Hu Y, Gao HJ (2011). Expression of PPARgamma and PTEN in human colorectal cancer: An immunohistochemical study using tissue microarray methodology. Oncol Lett.

[39] Chandrasekaran S, Deng H, Fang Y (2015). PTEN deletion potentiates invasion of colorectal cancer spheroidal cells through 3D Matrigel. Integr Biol (Camb).

[40] Zhang LL, Liu J, Lei S, Zhang J, Zhou W, Yu HG (2014). PTEN inhibits the invasion and metastasis of gastric cancer via downregulation of FAK expression. Cell Signal.

[41] Du K, Tsichlis PN (2005). Regulation of the Akt kinase by interacting proteins. Oncogene.

[42] Thanan R, Murata M, Ma N, Hammam O, Wishahi M, El Leithy T, Hiraku Y, Oikawa S, Kawanishi S (2012). Nuclear localization of COX-2 in relation to the expression of stemness markers in urinary bladder cancer. Mediators Inflamm.

[43] Parfenova H, Parfenov VN, Shlopov BV, Levine V, Falkos S, Pourcyrous M, Leffler CW (2001). Dynamics of nuclear localization sites for COX-2 in vascular endothelial cells. Am J Physiol Cell Physiol.

[44] Wilson JW, Potten CS (2000). The effect of exogenous prostaglandin administration on tumor size and yield in Min/+ mice. Cancer Res.

[45] Bol DK, Rowley RB, Ho CP, Pilz B, Dell J, Swerdel M, Kiguchi K, Muga S, Klein R, Fischer SM (2002). Cyclooxygenase-2 overexpression in the skin of transgenic mice results in suppression of tumor development. Cancer Res.

[46] Fleming ID (1997). American Joint Committee on Cancer., American Cancer Society. and American College of Surgeons. AJCC cancer staging manual.

[47] Richards CH, Roxburgh CSD, Anderson JH, McKee RF, Foulis AK, Horgan PG, McMillan DC (2011). Prognostic value of tumour necrosis and host inflammatory responses in colorectal cancer. The British journal of surgery.

[48] Richards CH, Roxburgh CS, Powell AG, Foulis AK, Horgan PG, McMillan DC (2014). The clinical utility of the local inflammatory response in colorectal cancer. European journal of cancer.

[49] Roxburgh CS, Richards CH, Macdonald AI, Powell AG, McGlynn LM, McMillan DC, Horgan PG, Edwards J, Shiels PG (2013). The *in situ* local immune response, tumour senescence and proliferation in colorectal cancer. British journal of cancer.

