# Supplementary material for: Relationship between tumour PTEN/Akt/COX-2 expression, inflammatory response and survival in patients with colorectal cancer
Source: Oncotarget. 2016 Sep 20;7(43):70601–12. doi: 10.18632/oncotarget.12134 (PMC5342577; doi:10.18632/oncotarget.12134)
Supplement: Supplementary file 1 [file oncotarget-07-70601-s001.pdf]

# Relationship between tumour PTEN/Akt/COX-2 expression, inflammatory response and survival in patients with colorectal cancer

## SUPPLEMENTARY FIGURE AND TABLE

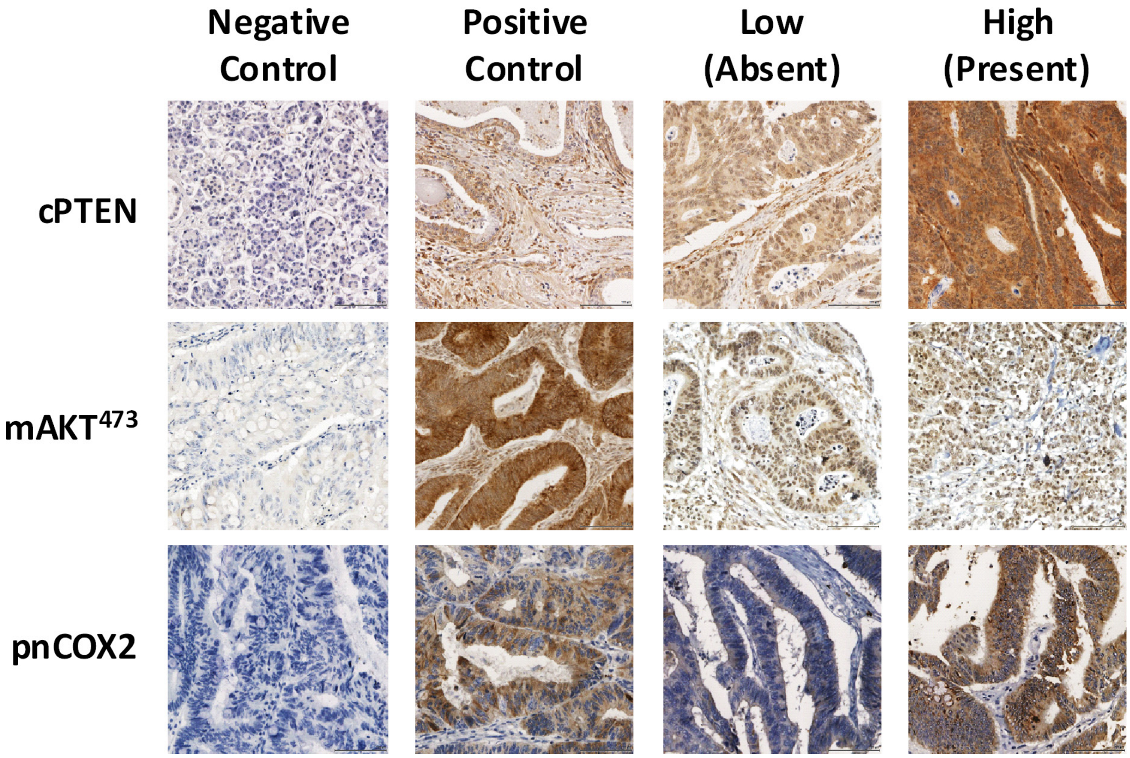


**Supplementary Figure 1: Immunohistochemical staining for cPTEN, mAkt$^{473}$ and nCOX2.** Representative images of staining for all three proteins are shown (x400). Negative and positive controls are also shown for each.

**Supplementary Table 1: Patient characteristics for CRC cohort (n=201)**

| Clinicopathological Characteristics | N (%) |
|---|---|
| **Age** | |
| <65 | 81 (40) |
| >65 | 120 (60) |
| **Sex** | |
| Female | 96 (48) |
| Male | 105 (52) |
| **Adjuvant** | |
| No | 145 (72) |
| Yes | 56 (28) |
| **Tumour site** | |
| Colon | 133 (66) |
| Rectum | 68 (34) |
| **TNM stage** | |
| 1 | 14 (7) |
| 2 | 96 (48) |
| 3 | 91 (45) |
| **Differentiation** | |
| Mod/well | 178 (89) |
| Poor | 23 (11) |
| **Venous invasion** | |
| Absent | 130 (65) |
| Present | 71 (35) |
| **Margin involvement** | |
| No | 194 (97) |
| Yes | 7 (3) |
| **Peritoneal involvement** | |
| No | 150 (75) |
| Yes | 51 (25) |
| **Mismatch repair status** | |
| Competent | 174 (87) |
| Deficient | 27 (13) |
| **Proliferation Index** | |
| Low | 72 (36) |
| High | 129 (64) |
| **Necrosis** | |
| Low | 122 (61) |
| High | 79 (39) |
| **Tumour stroma percentage (n=182)** | |
| Low | 140 (77) |
| High | 42 (23) |
| **Tumour Budding (n=186)** | |
| No | 126 (68) |
| Yes | 60 (32) |
